# The impact of glucose metabolism on inflammatory processes in sepsis-induced acute lung injury

**DOI:** 10.3389/fimmu.2024.1508985

**Published:** 2024-12-06

**Authors:** Shilei Cheng, Yufei Li, Xiaoliang Sun, Zhirui Liu, Liang Guo, Jueheng Wu, Xiaohan Yang, Sisi Wei, Guanghan Wu, Shilong Xu, Fan Yang, Jianbo Wu

**Affiliations:** ^1^ School of Anesthesiology, Shandong Second Medical University, Weifang, China; ^2^ Department of Anesthesiology, The First Affiliated Hospital of Shandong First Medical University & Shandong Provincial Qianfoshan Hospital, Jinan, China; ^3^ Shandong Institute of Anesthesia and Respiratory Critical Medicine, Jinan, China; ^4^ Shandong Provincial Clinical Research Center for Anesthesiology, Jinan, China; ^5^ School of Pharmacy, Shandong University of Traditional Chinese Medicine (TCM), Jinan, China; ^6^ Department of Urology, Shandong Provincial Hospital Affiliated to Shandong First Medical University, Jinan, China; ^7^ Brain and Mind Centre, Faculty of Medicine and Health, The University of Sydney, Sydney, NSW, Australia; ^8^ Department of Anesthesiology, Qilu Hospital of Shandong University Dezhou Hospital, Dezhou, China

**Keywords:** sepsis, ALI, glycolysis, oxidative phosphorylation (OXPHOS), metabolic reprogramming, immune response

## Abstract

Acute lung injury (ALI) is a prevalent and critical complication of sepsis, marked by high incidence and mortality rates, with its pathogenesis still not being fully elucidated. Recent research has revealed a significant correlation between the metabolic reprogramming of glucose and sepsis-associated ALI (S-ALI). Throughout the course of S-ALI, immune cells, including macrophages and dendritic cells, undergo metabolic shifts to accommodate the intricate demands of immune function that emerge as sepsis advances. Indeed, glucose metabolic reprogramming in S-ALI serves as a double-edged sword, fueling inflammatory immune responses in the initial stages and subsequently initiating anti-inflammatory responses as the disease evolves. In this review, we delineate the current research progress concerning the pathogenic mechanisms linked to glucose metabolic reprogramming in S-ALI, with a focus on the pertinent immune cells implicated. We encapsulate the impact of glucose metabolic reprogramming on the onset, progression, and prognosis of S-ALI. Ultimately, by examining key regulatory factors within metabolic intermediates and enzymes, We have identified potential therapeutic targets linked to metabolic reprogramming, striving to tackle the inherent challenges in diagnosing and treating Severe Acute Lung Injury (S-ALI) with greater efficacy.

## 1 Introduction

Sepsis, a grave condition stemming from a dysregulated host response to infection, leads to life-threatening organ dysfunction. It is categorized as a critical clinical scenario with an alarmingly high mortality rate, standing as a principal and direct cause of death related to infections. Globally, sepsis accounts for approximately 48.9 million cases annually, culminating in nearly 11 million fatalities, positioning it as the preeminent cause of mortality among patients in intensive care units (1, 2).

The lungs, being particularly vulnerable, are the most commonly targeted organs in sepsis. Acute lung injury (ALI) and acute respiratory distress syndrome (ARDS) are among the most frequent complications of sepsis, representing major causes of short-term mortality and long-term deterioration in quality of life for patients (3). Research suggests that around 50% of sepsis patients develop ALI or ARDS, which are associated with a staggering 40% mortality rate (4, 5). Clinical manifestations of sepsis-induced lung injury are predominantly characterized by persistent hypoxemia and respiratory distress (6, 7). Pathologically, this involves damage to the pulmonary vascular endothelium, diminished alveolar surface tension, the release of inflammatory mediators, and interstitial fibrosis. The precise mechanisms behind lung injury in sepsis remain elusive, but current theories propose that they may involve the disruption of the endothelial barrier, microthrombosis, an imbalance in the inflammatory response, cellular apoptosis and necrosis, immune dysregulation, mitochondrial dysfunction, and metabolic disturbances (8, 9). The Third International Consensus on Sepsis and Septic Shock (Sepsis 3.0) emphasizes the importance of the host’s metabolic derangements and the systemic response imbalance in defining sepsis (10).

**Figure 1:**
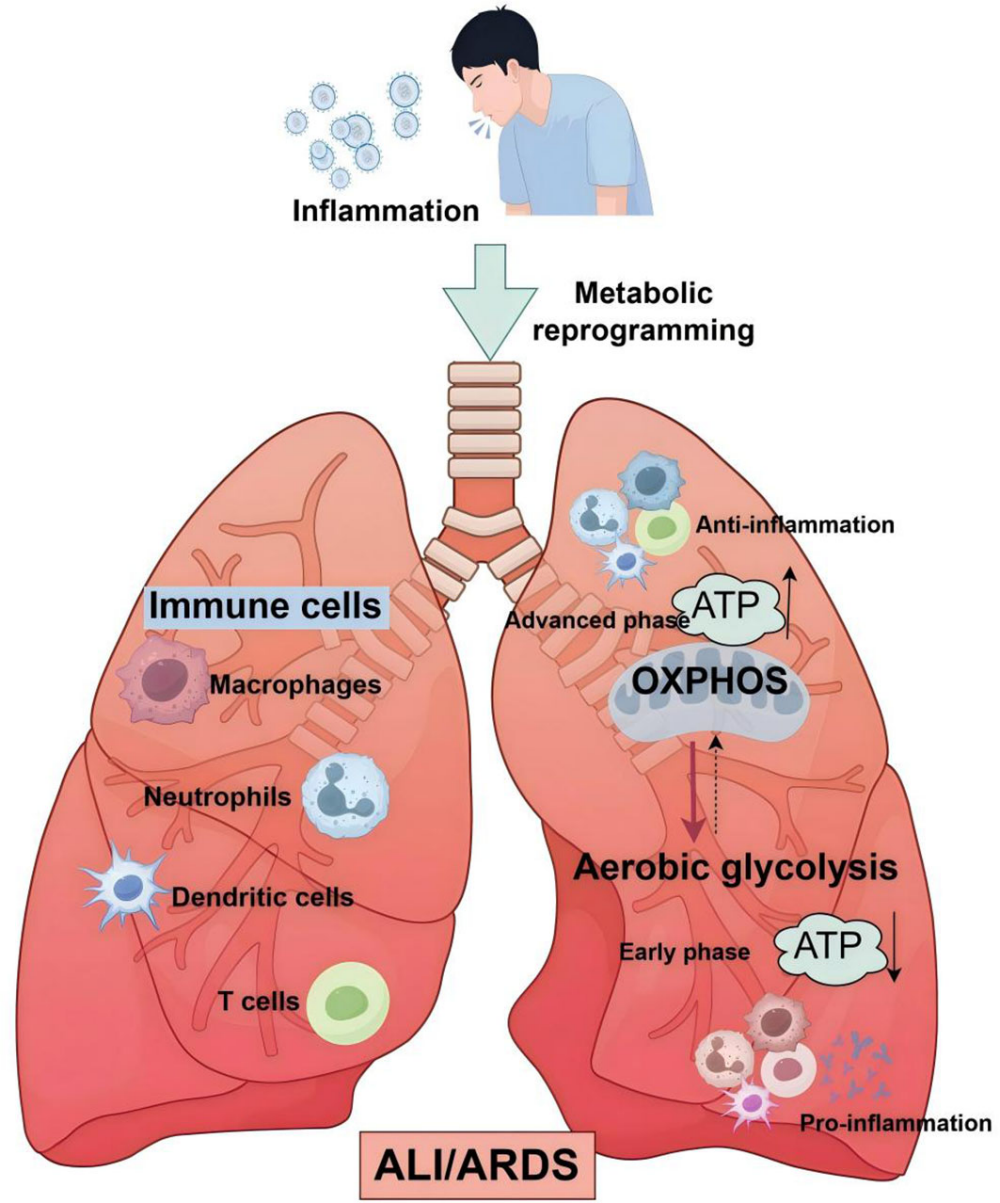
Glycolytic reprogramming in immune cells: a pivotal factor in ALI/ARDS development. In the initial phases of sepsis, immune cells, notably macrophages, are swiftly activated in response to stimuli and unleash a surge of inflammatory mediators and cytokines. Concurrently, there is a significant shift in the primary energy metabolism of these immune cells, transitioning from the slow, energy-efficient oxidative phosphorylation to the rapid, yet energy-limited, aerobic glycolysis. This metabolic switch is crucial for understanding the pathogenesis of Acute Lung Injury (ALI) and Acute Respiratory Distress Syndrome (ARDS).

A multitude of studies have validated that sepsis triggers widespread disruptions and imbalances across multiple systems, encompassing the immune, respiratory, urinary, endocrine, and nervous systems (11–13). Metabolic disorders, characterized by aberrations or imbalances in metabolic processes, disrupt the biochemical equilibrium and thus impair normal physiological functions. In the context of sepsis-induced lung injury, these metabolic disturbances encompass a range of biochemical pathways, including glucose, lipid, protein metabolism, and trace element metabolism (14–17). Immunometabolism has emerged as a focal point of research, with mounting evidence suggesting a close link between glucose metabolism reprogramming and immune cell activation. As shown in **Figure 1**, there is a significant shift in the primary energy metabolism of these immune cells, transitioning from the slow, energy-efficient oxidative phosphorylation to the rapid, yet energy-limited, aerobic glycolysis. This article delves into the pathogenesis of sepsis-associated acute lung injury (S-ALI) through the lenses of immunity and metabolism. As immunometabolomics research gains momentum, it has revealed the intricate interplay between inflammatory responses and metabolic alterations. For instance, compounds such as nivanone, solanolide A, and baicalin (NP) mitigate hyperimmunity in ALI by targeting the leucine/PI3K/Akt/mTOR signaling cascade (18). Additionally, immune cells like macrophages and lymphocytes undergo substantial metabolic reprogramming and distinct epigenetic modifications post-activation during ALI. These metabolic changes not only provide ATP and biosynthetic precursors but also modulate inflammatory and immune responses (19). To elucidate the role of metabolic pathway modulation in S-ALI, we harness the power of immunometabolic profiling, which aids in identifying alterations in key metabolic pathways and metabolites within lung, serum, and fecal tissues. These changes may be involved in the occurrence and development of ALI and provide potential therapeutic targets (20). Consequently, comprehending how adverse factors in ALI impact the immune metabolism of differentiated immune cells can lay the groundwork for discovering new metabolic intermediates and therapeutic targets for enzymes, as well as tackling the challenges posed by immune metabolism in ALI (21). Mirroring the Warburg effect observed in cancer cells, both immune and non-immune cells undergo metabolic reprogramming following sepsis. However, the precise mechanisms by which inflammation influences specific cells remain partially understood. This review encapsulates the advancements in research on glucose metabolism reprogramming in sepsis, particularly within the context of ARDS, and explores glucose metabolism-based strategies for ARDS treatment. It provides novel insights into the pathogenesis and therapeutic approaches for sepsis.

## 2 The relationship between glucose metabolism and sepsis

Traditional perspectives posit that the alterations in cellular glucose metabolism and the onset of hyperlactatemia observed in sepsis, particularly when accompanied by pulmonary injury, are primarily due to a deficiency in oxygen delivery to tissue cells (22, 23). However, ongoing research suggests that hypoxia alone is insufficient to explain the comprehensive and systemic metabolic perturbations encountered in sepsis. Furthermore, despite contemporary therapeutic strategies that maintain sufficient oxygenation for sepsis patients, cellular metabolic alterations continue to be observed, prompting a growing consensus among researchers that sepsis itself may be a catalyst for changes in cellular metabolic pathways. Recent studies have revealed that sepsis triggers a metabolic reprogramming in the majority of cells, with immune cells exhibiting metabolic shifts reminiscent of those seen in cancer cells, notably a preference for aerobic glycolysis, a phenomenon commonly referred to as the “Warburg effect” (24–26). This insight offers fresh avenues for the exploration of the pathogenesis and potential therapeutic approaches to sepsis-induced lung injury.

**Figure 2:**
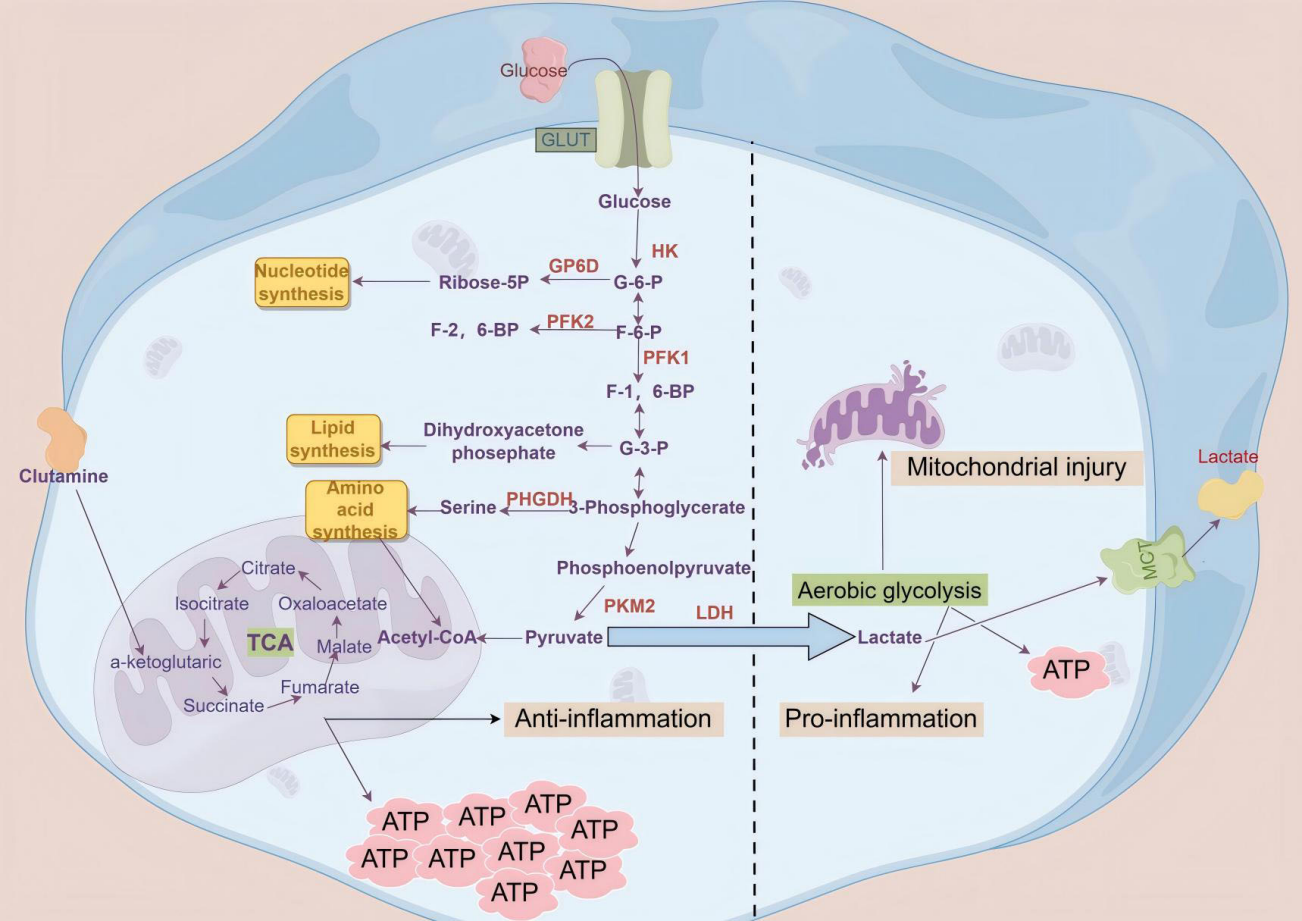
Schematic diagram of oxidative phosphorylation and aerobic glycolysis cellular glycolysis begins when glucose enters the cell through glucose transporters on the cell membrane. In the cytoplasm, glucose is broken down into two molecules of pyruvate via the glycolytic pathway, which then enter the mitochondria. Through the tricarboxylic acid cycle and oxidative phosphorylation, a large amount of ATP and water is generated, releasing carbon dioxide. However, during sepsis, cells may experience mitochondrial dysfunction due to inflammation and oxidative stress, leading to a reliance on glycolysis to meet energy demands even in the presence of sufficient oxygen, resulting in the excessive production and abnormal accumulation of lactate.

The Warburg effect, alternatively termed aerobic glycolysis or the metabolic reprogramming of glucose metabolism, describes the distinctive metabolic behavior observed in cancer cells. Unlike their normal counterparts, these cells preferentially convert the majority of pyruvate, the end product of glycolysis, into lactate and secrete it from the cell. This process bypasses the complete oxidation of pyruvate in the mitochondria through the citric acid cycle and oxidative phosphorylation, which would otherwise yield a substantial amount of ATP (27). Under aerobic conditions, mature immune cells paradoxically rely on glycolysis for ATP production, even in the absence of proliferation following activation. The majority of pyruvate produced from glycolysis is diverted towards lactate conversion, while glycolytic intermediates accumulate to satisfy the cells’ heightened energy requirements. Glycolysis not only supplies the ATP necessary for these cells but also provides glucose-6-phosphate, a key substrate for nucleotide synthesis. However, upon stimulation, immune cells such as activated macrophages or dendritic cells (DCs) transition from oxidative phosphorylation (OXPHOS) to glycolysis, a metabolic shift reminiscent of the “Warburg effect” which is shown in **Figure 2**. This is triggered by various stimuli, including lipopolysaccharide (LPS), the TLR3 ligand poly(I:C), and type I interferons (IFNs). During this shift, there is a notable reduction in TCA cycle activity, accompanied by an increase in lactate production and pentose phosphate pathway (PPP) flux. The augmented PPP activity facilitates the synthesis of purines and pyrimidines, essential for the biosynthesis of activated cells, and also generates NADPH, which is utilized by NADPH oxidases to produce reactive oxygen species (ROS). This metabolic reorientation towards heightened glycolysis may facilitate rapid ATP generation. Although less efficient than the TCA cycle in ATP production, this pathway can be quickly activated and may be particularly advantageous for ATP synthesis in cells with a high capacity for glucose uptake (28–32).

## 3 Effector cells involved in glucose metabolism reprogramming in sepsis

### 3.1 Immune cells

The immune dysregulation that occurs during sepsis is intricate, encompassing both an overexuberant immune response and a concurrent suppression of immune function. Initially, sepsis is marked by an exaggerated activation of the immune system in response to infection, resulting in the profuse release of inflammatory mediators and cytokines. However, as the condition advances, patients often transition into a state of immune suppression, evidenced by diminished lymphocyte counts—particularly a decline in CD4^+^ and CD8^+^ T cells—alongside a reduction in immune cell functionality and an upregulation of regulatory T cells (Tregs). This suppression heightens the risk of secondary infections, potentially exacerbating the severity of the disease. Investigations into immune dysregulation associated with sepsis have established that the activation or suppression of immune cells is intricately linked to alterations in their energy metabolism. Glycolysis swiftly generates energy to satisfy the heightened demands of immune cells and produces metabolic byproducts that facilitate the synthesis of crucial biological intermediates, thereby supporting immune cell proliferation and differentiation. As a result, glycolysis emerges as the predominant energy metabolism pathway for most immune cells when activated. Consequently, the metabolic shift towards aerobic glycolysis is advantageous for immune system activation during sepsis, bolstering its capacity to eliminate pathogens (33–35). Changes in the metabolic pathways of immune cells play a crucial role in maintaining immune cell function and controlling immune homeostasis (**Table 1**).

**Table 1:** Key immune cells involved in the process of septic lung injury.

Cells	Classifications	Primary energy supply mechanism	Functions	References
Macrophages	M0, M1, M2	M1 primarily utilizes OXPHOS as the metabolic pathway, while M2 mainly relies on glycolysis.	M1 pro-inflammatory effects, M2 anti-inflammatory effects.	(36, 37)
Neutrophils	/	Glycolysis	Formation of NETs exerts pro-inflammatory effects.	(38, 39)
Dendritic Cells	Resting state	OXPHOS	Immune reserve	(40–42)
	Active state	Glycolysis	Pro-inflammatory	
T lymphocytes	Immature T lymphocytes	OXPHOS	Immune reserve	(43–45)
	Memory T cells	OXPHOS	Rapid activation to produce an immune response.	
	Effector T cells (Th1, Th2, Th12)	Glycolysis	Pro-inflammatory	
	Regulatory T cells	OXPHOS and FAO	Anti-inflammatory	
B lymphocytes	Resting state	Low metabolic level	Immune reserve	(46, 47)
	Active state	Glycolysis	Humoral immunity	
NK cells	Resting state	Low metabolic level	Immune reserve	(48, 49)
	Active state	Both glycolysis and OXPHOS are upregulated	Cytotoxic effect	

#### 3.1.1 Macrophages

Extensive research has substantiated that sepsis is linked to a spectrum of alterations in glucose metabolism. Macrophages, which are pivotal in both the innate and adaptive immune responses, act as the frontline of defense within the immune system. They are instrumental in combating pathogen incursions and in preserving homeostatic balance (50). Ubiquitous throughout the body, macrophages display considerable heterogeneity and plasticity, adapting their functions in response to diverse danger signals. These signals include pathogen challenges or indications of tissue damage, which prompt macrophages to differentiate into either pro-inflammatory M1 phenotypes or anti-inflammatory M2 phenotypes. They are central to the sepsis-related lung injury, playing a critical part in both the initial excessive inflammatory response and the subsequent immunosuppressive phase (51, 52). Meanwhile, an increasing number of studies have confirmed that macrophage activation is closely related to the reprogramming of glucose metabolism (53). Under resting conditions, macrophages primarily rely on mitochondrial oxidative phosphorylation for energy production (54). Upon activation and polarization towards the M1 phenotype by stimuli such as interferon-γ (IFN-γ), interleukin-1β (IL-1β), and lipopolysaccharide (LPS), macrophages undergo a metabolic shift. Aerobic glycolysis supplants the tricarboxylic acid (TCA) cycle as the predominant pathway for ATP generation, fulfilling the rapidly escalating energy requirements of these activated cells. Moreover, intermediates of glycolysis can serve as precursors for the synthesis of inflammatory factors in M1 macrophages, contributing to the inflammatory response (36). Research has demonstrated that the elevated expression of genes associated with aerobic glycolysis, such as hexokinase (HK), phosphofructokinase-1 (PFK1), glucose transporter 1 (GLUT1), lactate dehydrogenase A (LDHA), and pyruvate dehydrogenase kinase 1 (PDK1), is a significant contributor to the enhanced glycolytic activity observed in M1 macrophages (55). The regulation of PFK1 activity is predominantly governed by phosphofructokinase 2 (PFK2). In the context of sepsis, there is a transition of PFK2 from the less active L-type, which is encoded by the fructose-2,6-bisphosphatase 1 (PFKFB1) gene, to the more potent U-type, encoded by the fructose-2,6-bisphosphatase 3 (PFKFB3) gene. This metabolic switch results in an increased production of fructose-2,6-bisphosphate, thereby augmenting glycolytic flux, elevating lactate levels, and facilitating the release of high mobility group box 1 protein (HMGB1) and interleukin-1β (IL-1β) (56). Furthermore, research has identified two critical metabolic checkpoints within the tricarboxylic acid (TCA) cycle of M1 macrophages, specifically at the levels of isocitrate dehydrogenase and succinate dehydrogenase. These regulatory points diminish the efficiency of the mitochondrial oxidative respiratory chain and disrupt the flow of the TCA cycle, leading to the accumulation of citrate and succinate (57). Citrate, in the cytoplasm, is cleaved by ATP-citrate lyase into acetyl-CoA and oxaloacetate. Acetyl-CoA, in turn, acts as a substrate for fatty acid synthesis and is instrumental in the generation of prostaglandins and nitric oxide (NO), both of which play significant roles in inflammatory responses. Meanwhile, succinate interacts with hypoxia-inducible factor-1α (HIF-1α) and the promoter region of the IL-1β gene, thereby enhancing the production of IL-1β and driving further inflammation (58).

M2 macrophages are predominantly activated by interleukin-4 (IL-4) and interleukin-13 (IL-13) (59). These macrophages possess a fully functional tricarboxylic acid (TCA) cycle, which allows them to satisfy their energy requirements primarily through the oxidative phosphorylation of glucose in the mitochondria. Additionally, they are capable of harnessing fatty acid oxidation (FAO) as an energy source, but they typically do not depend on aerobic glycolysis for their metabolic needs (37). This metabolic profile is largely attributable to the ability of M2 macrophages to convert PFK2 to the L-type by overexpressing PFKFB1. This enzyme degrades the glycolytic inducer fructose-2,6-bisphosphate into fructose-6-phosphate, consequently lowering the rate of glycolysis (60). M2 macrophages mitigate tissue and cellular inflammation by secreting anti-inflammatory cytokines such as interleukin-10 (IL-10) and transforming growth factor-beta (TGF-β). Additionally, they overexpress anti-inflammatory genes like arginase (Arg)-1 and CD206, which are crucial for tissue repair and reconstruction during the resolution phase of inflammation (61, 62).

Specifically, alveolar macrophages (AMs), as the resident macrophages of the airway, serve as the frontline immune sentinels of the respiratory tract. Beyond their established role in host defense, AMs are pivotal in orchestrating inflammatory responses and in preserving the integrity of respiratory tissues (63, 64). However, it is important to recognize that alveolar macrophages (AMs) reside in environments with notably low glucose concentrations, rendering glycolysis-based immune metabolism an inefficient and improbable pathway for immune activation. Several studies have corroborated that AMs do not undergo heightened glycolysis in the early stages of sepsis-induced lung injury. Moreover, the inhibition of glycolysis has been found to have no impact on their proinflammatory cytokine production. Instead, AMs predominantly rely on oxidative phosphorylation (OXPHOS) to generate cytokines during lipopolysaccharide (LPS)-induced activation (65, 66). When ALI develops into ARDS, AMs are exposed to extreme hypoxia, and the metabolic state of AMs will change to glycolysis under the induction of HIF-1α activation (67–69). Consequently, as the most critical macrophages within the pulmonary system, the activation of alveolar macrophages can trigger the activation of other immunocompetent cells and the subsequent release of inflammatory mediators. This chain of events can lead to an uncontrolled local inflammatory response in the lungs, severely impacting the prognosis of sepsis-induced lung injury (70, 71). Therefore, regulating macrophage glucose metabolism to influence their polarization presents a promising new approach for the treatment of sepsis-induced lung injury.

#### 3.1.2 Neutrophils

Neutrophils constitute the majority of white blood cells in the immune system, making up roughly 50% to 70% of the total leukocyte count in peripheral blood. They are widely regarded as terminally differentiated effector cells (72, 73). Neutrophils can quickly detect and eliminate pathogenic microorganisms in the body, and their abnormal activation is considered one of the markers of septic lung injury (74–76). In the context of septic lung injury, there is a significant accumulation of neutrophils within the lungs. This influx augments the release of inflammatory cytokines, compromises the integrity of both the epithelial and endothelial barriers, and fosters the progression of interstitial pulmonary edema (77). The function of neutrophils typically relies on cytoskeletal remodeling and energy supply, with the traditional view being that the energy for mature neutrophils primarily comes from glycolysis (38). Additional research indicates that mitochondrial oxidative phosphorylation plays a crucial role in neutrophil motility. Inhibition of mitochondrial ATP production or mTOR activation can significantly impair the chemotactic ability of neutrophils (25, 78). These insights imply that both glycolysis and mitochondrial respiration are pivotal in facilitating neutrophil motility. Neutrophils navigate to damaged tissues via migration and modulate inflammatory responses through a variety of mechanisms, including phagocytosis, degranulation, and the formation of Neutrophil Extracellular Traps (NETs) (39). Neutrophil Extracellular Traps (NETs), rich in granule proteins modified by histones, can exacerbate sepsis by fueling the initiation and escalation of inflammatory responses. A study involving sepsis patients with ARDS revealed that plasma NET levels were positively correlated with the severity of ARDS and mortality rates. Moreover, therapeutic intervention with DNase I, an enzyme that degrades NETs, has been shown to decrease NET production and mitigate lung injury in mouse models of severe bacterial pneumonia and acute lung injury (79–82). Furthermore, research has demonstrated that in both sepsis-induced lung injury patients and corresponding mouse models, NETs trigger the METTL3-mediated m6A-IGF2BP2-dependent pathway, resulting in the upregulation of HIF-1α. This leads to an intensification of glycolysis and a suppression of oxidative phosphorylation in alveolar epithelial cells, thereby exacerbating ferroptosis within these cells and playing a pivotal role in the progression of sepsis-induced lung injury (83). As the body gradually recovers, neutrophils primarily initiate clearance mechanisms through apoptosis, and neutrophil apoptosis is inversely correlated with the severity of sepsis (84). Consequently, neutrophils display distinct physiological states across the various stages of sepsis development, indicating that they assume unique temporal and spatial roles within this complex process.

#### 3.1.3 Dendritic cells

Dendritic cells (DCs), as antigen-presenting cells, also participate in coordinating the body’s innate and adaptive immune responses during inflammatory reactions in sepsis-induced lung injury (85, 86). Like macrophages, dendritic cells (DCs) predominantly depend on mitochondrial oxidative phosphorylation for energy when in their resting state. Upon detection of homeostatic perturbations instigated by inflammatory signals, DCs shift from a quiescent to an activated state, undergoing a suite of metabolic alterations to modulate their survival and immune functions, with a notable upregulation of aerobic glycolysis (40–42).

HIF-1α is a pivotal molecule in the activation of mature dendritic cells, playing a crucial role in both glycolysis and the release of pro-inflammatory cytokines. It stimulates and enhances the activity of pyruvate kinase M2, which in turn leads to the upregulation of key rate-limiting glycolytic enzymes, such as hexokinase and phosphofructokinase. This cascade of events results in a significant boost to glycolytic activity (87). Research has illuminated that lipopolysaccharide (LPS) prompts an upregulation of pyruvate dehydrogenase kinase 2 (PDK2) in dendritic cells via the activation of Toll-like receptors (TLRs). The overexpression of PDK2 intensifies the activation of the TLR4 signaling pathway, which in turn induces the production of pro-inflammatory cytokines in dendritic cells and leads to metabolic reprogramming. On the flip side, the downregulation of PDK2 expression dampens TLR4 signaling, markedly reducing mortality in septic mice models and mitigating pulmonary pathology (88, 89).

Furthermore, the metabolic trajectory of LPS-activated dendritic cells is contingent upon the activity of inducible nitric oxide synthase (iNOS) and nitric oxide (NO)-mediated mitochondrial metabolism. Upon the initiation of TLR4 signaling, the expression of iNOS is heightened. iNOS catalyzes the synthesis of NO by combining oxygen radicals with nitrogen atoms sourced from arginine. This NO production inhibits the mitochondrial electron transport chain through a process of nitrosylation, consequently suppressing oxidative phosphorylation (OXPHOS) and concurrently promoting glycolysis in dendritic cells (90). Studies reveal that microRNA-142 (miR-142) orchestrates the metabolic shift in dendritic cells by targeting the pivotal enzyme in fatty acid oxidation, carnitine palmitoyltransferase-1a (CPT1a). In the absence of miR-142, there is an escalation in fatty acid oxidation, which in turn promotes the expression of IL-10. This favors oxidative phosphorylation (OXPHOS) and impedes the dendritic cells’ capacity to switch from OXPHOS to glycolysis, a transition that is crucial for their metabolic reprogramming (91, 92).

#### 3.1.4 T lymphocytes

T lymphocytes, as a major type of adaptive immune cell, play a crucial role during sepsis-induced lung injury (93, 94). Immature T lymphocytes primarily rely on mitochondrial oxidative phosphorylation and fatty acid oxidation for energy (95, 96). When dendritic cells, as key antigen-presenting cells, display antigens to naïve T lymphocytes, these T cells are stimulated to activate, subsequently undergoing clonal expansion and differentiation. Distinct T lymphocyte subsets demonstrate unique metabolic signatures that are integral to their rapid clonal proliferation, differentiation, and functional execution (97). Memory T cells (Tmems) swiftly activate to mount immune responses, primarily harnessing oxidative phosphorylation (OXPHOS) for their energy needs. Effector T cells (Teffs), including Th1, Th2, and Th17 subsets that predominantly mediate pro-inflammatory actions, rely on glycolysis for energy production. Concurrently, regulatory T cells (Tregs) exhibit a unique metabolic signature compared to their effector counterparts. For the differentiation of activated T lymphocytes into Tregs, glycolytic activity must be curtailed, necessitating a metabolic pivot from glycolysis to OXPHOS, with fatty acid oxidation (FAO) and OXPHOS emerging as the principal ATP sources. Nonetheless, this shift does not diminish the significance of glycolysis in the function of Tregs (43–45). Inhibition of glycolysis not only diminishes the anti-inflammatory activity of Tregs but also suppresses the expression of forkhead box P3 (Foxp3) through enolase-1 (ENO1). Foxp3, a specific transcription factor indispensable for Treg differentiation, exerts a pivotal regulatory influence on the metabolic pathways of T lymphocytes. It does so by bolstering oxidative phosphorylation (OXPHOS) and curbing glycolysis, thereby maintaining the balance between these metabolic processes (98, 99).

Aerobic glycolysis is an essential metabolic pathway for the activation of T lymphocytes. The phosphoinositide 3-kinase (PI3K)/protein kinase B (Akt)/mammalian target of rapamycin (mTOR) signaling cascade plays a central role in this process by activating hypoxia-inducible factor-1α (HIF-1α) and the transcription factor Myc. These activations, in turn, stimulate aerobic glycolysis and lactate dehydrogenase expression in effector T lymphocytes, thereby facilitating their metabolic and functional demands (100). The mTOR signaling node is comprised of two distinct complexes, mTORC1 and mTORC2. CD4+ T cells deficient in mTORC1 are impaired in their ability to differentiate into the Th1, Th2, or Th17 lineages. Th17 cells are particularly influential in the development and progression of sepsis, and the balance between Th17 cells and Tregs has a direct impact on the trajectory and resolution of inflammatory responses (101). Th17 cells are elevated during the initial stages of sepsis, but as the condition advances into its later stages, there is an increase in Th17 cell apoptosis, leading to a reduction in their population. This decline contributes to immune paralysis or suppression and is significantly associated with a higher mortality rate among sepsis patients (101). The PI3K/Akt/mTORC1/ribosomal protein S6 kinase 1 (S6K1) axis inhibits Gfi1, regulating Th17 cell generation. Additionally, S6K1 binds to the retinoic acid receptor-related orphan nuclear receptor gamma t (RORγt) and transports it into the nucleus, inducing gene expression related to Th17 cell functional phenotypes (102). Studies have revealed that itaconate, functioning as an immunoregulatory metabolite, plays a pivotal role in modulating the equilibrium between Th17 and Treg cells. It achieves this by dampening glycolysis and oxidative phosphorylation in polarized T lymphocytes, thus orchestrating the balance of Th17 to Treg cell ratios through metabolic and epigenetic reprogramming (103). Furthermore, as a burgeoning area of research, lactate has been shown to induce dysregulation of specific gene expression programs in Th17 cells by modulating their metabolic pathways, effectively reprogramming pro-inflammatory T cell phenotypes towards a regulatory T cell profile. Th17 cells treated with lactate exhibit increased reactive oxygen species (ROS) production in mitochondria, which in turn stimulates IL-2 secretion and enhances Foxp3 expression. Mechanistically, this is accompanied by an elevated global level of H3K18 lactylation in Th17 cells, suggesting that lactate can influence the epigenetic landscape of Th17 cells through metabolic regulation (104, 105). Furthermore, metabolic reprogramming and dysregulated IL-17 production persist after sepsis resolution, impairing CD4+ T cell function and increasing the risk of infection-related readmission and mortality in sepsis survivors (106). These discoveries present novel avenues and therapeutic strategies for addressing the Th17/Treg cell imbalance in the management of sepsis-induced lung injury.

It is widely accepted that during the inflammatory response in sepsis-induced lung injury, glycolysis fuels inflammation, while oxidative phosphorylation (OXPHOS) underpins anti-inflammatory processes. Upon the activation of sepsis-related immune cells, there is a metabolic shift from OXPHOS to aerobic glycolysis to satisfy the heightened energy demands associated with immune cell activation. However, an overreliance on aerobic glycolysis by immune cells over an extended period can result in immune suppression, which in turn impacts the prognosis of sepsis. Thus, modulating the metabolic reprogramming of immune cells is of paramount importance in the treatment of sepsis-induced lung injury. Unraveling the potential role of glycolytic reprogramming in immune cells during sepsis is crucial for deepening our understanding of the pathophysiology of sepsis and may pave the way for innovative therapeutic interventions.

#### 3.1.5 B lymphocytes

B lymphocytes, integral to the realm of humoral immunity, are capable of producing immunoglobulins upon appropriate stimulation. They also play a role in amplifying cytokine responses and facilitating bacterial clearance through their interactive communication with other immune cells, including macrophages (107, 108). In the context of sepsis, the humoral immune response necessitates swift biosynthesis, proliferation, and differentiation of B cells. These processes entail significant phenotypic transformations in B cells, which, coupled with substantial metabolic shifts, accommodate the dramatically altered energy requirements as B cells evolve from a dormant state to an activated, vigorously proliferative one (109). Naive B cells maintain a state of quiescence, characterized by a minimal basal metabolic rate. Upon exposure to heightened infectious challenges, this equilibrium is disrupted, with a notable escalation in aerobic glycolysis and mitochondrial oxidative phosphorylation (OXPHOS). This metabolic shift has been predominantly linked to the upregulation of key glycolytic molecules, such as GLUT1 and LDHA, which is orchestrated by the activation of B cell receptors (46, 47). To sustain this heightened metabolic activity and to avert mitochondrial dysfunction and the ensuing cell death that can accompany activation, an additional signal is imperative. Such signals may include those derived from T helper cells or those transduced through Toll-like receptor 9 (TLR9) (110). However, research has indicated that during sepsis, there is a decrease in the levels of human leukocyte antigen DR (HLA-DR) on B cells, which diminishes their capacity to engage with T helper cells, thereby depriving them of the necessary second signal. Moreover, there is an exacerbation of proton leak, which contributes to mitochondrial dysfunction (111). These findings imply that B cell activation during sepsis is not sustainable. This notion of unsustainable B cell activation is corroborated by multiple studies utilizing mouse sepsis models, which document a diminished capacity for antigen-specific humoral immune responses in sepsis. This reduction in humoral immunity contributes to the onset of immunosuppression (112, 113). Consequently, the reduction in B cell numbers and the impairment of their functionality are regarded as indicative biomarkers for the progression of sepsis, reflecting its worsening course (114–116). Intriguingly, recent research has revealed that the glycolytic byproduct lactate can stimulate the proliferation of B cells. This can occur either by augmenting the expression of angiogenin (ANG), which facilitates the cleavage of the anticodon loop in mature tRNA, or by inducing the expression of miR-223, which targets the Fbw7 gene (117–119). It has become increasingly clear that the interplay between lactate and immune cells is pivotal for comprehending the immunodysregulation that accompanies sepsis. This understanding is essential for guiding future research endeavors and for refining therapeutic strategies aimed at optimizing patient outcomes.

#### 3.1.6 Natural killer cells

Natural Killer (NK) cells are classified within the ILC1 subset of Innate Lymphoid Cells (ILCs). Constituting the vanguard of the body’s immune defense, NK cells possess the innate ability to swiftly eliminate virus-infected cells and tumor cells without the need for prior sensitization (120). In contrast to B cells and T cells, NK cells lack the ability to recognize antigens specifically. Instead, their responsiveness to pathogens or their tolerance is governed by the intricate balance of signals emanating from a diverse array of activating and inhibitory receptors, which are stochastically combined (121). Studies have shown that the metabolic activity of NK cells is closely related to their activation status (122, 123). Under homeostatic conditions, murine NK cells display a metabolically quiescent phenotype, characterized by relatively low levels of glycolysis and oxidative phosphorylation. This inactive yet efficient metabolic profile is adequate to fulfill their biosynthetic and energy requirements. Fascinatingly, upon short-term activation by cytokines such as IL-12 and IL-15, or by NK receptors (NKRs) including NK1.1 and Ly49D, these cells can execute their effector functions without significantly changing their metabolic rates (48, 124). Conversely, in the later stages of sepsis-associated ALI/ARDS, sustained activation augments the expression of glycolytic enzymes and nutrient transporters. This leads to an upregulation of both glycolysis and oxidative phosphorylation (OXPHOS), ensuring a steady energy supply in response to continuous viral antigen stimulation and, consequently, enhancing their cytotoxic capabilities (48, 124–127). Glycolysis and oxidative phosphorylation (OXPHOS) are both crucial metabolic pathways for NK cell function. However, it has been observed that during prolonged activation, NK cells exhibit a preference for glycolytic metabolism, which can supply energy more swiftly. This preference may arise from the upregulation of glycolytic enzyme genes, a response to the deficiency in oxidative phosphorylation caused by the absence of cyclooxygenase-2 (COX2). These insights suggest that NK cells are adept at swiftly adapting to metabolic changes to preserve their functional integrity (128). Ultimately, if stimulation endures over the long term, NK cells may progress into an exhausted phenotype. This state is marked by metabolic dysfunction, diminished cytotoxicity, and an upregulation of surface inhibitory receptors (123, 129). This transition is attributed to the accumulation of high levels of lactate from glycolysis, which induces mitochondrial dysfunction and apoptosis in NK cells. Under such circumstances (119, 130), it often leads to a worsening of the prognosis for patients with sepsis-associated ALI/ARDS

### 3.2 Nonimmune cells

In the context of sepsis-induced acute lung injury, alongside the metabolic reprogramming of immune cells that modulates the body’s inflammatory response, there is a significant alteration in the function and metabolic state of non-immune cells, such as pulmonary vascular endothelial cells and epithelial cells. These cells are pivotal in preserving the lung’s normal architecture and physiological function. Recent research has increasingly demonstrated that non-immune cells contribute not only to structural integrity but also engage in the modulation of local and systemic inflammatory responses. The reprogramming of glucose metabolism, in particular, is regarded as a critical adaptive mechanism by which these cells respond to the dynamic changes within the septic microenvironment (131, 132). In the sepsis state, pulmonary vascular endothelial cells and epithelial cells augment their adaptability to the inflammatory milieu by altering their energy metabolism, which in turn impacts the stability of the microenvironment and the extent of cellular damage. Consequently, an in-depth investigation into the specific mechanisms underlying glucose metabolic reprogramming in non-immune cells during sepsis-induced acute lung injury could yield novel insights and therapeutic targets to enhance clinical treatment strategies.

#### 3.2.1 Endothelial cells

Endothelial cells (ECs) constitute a unique and multifunctional cell lineage. Paramount among their roles is serving as a semipermeable barrier between circulating blood and the surrounding tissues. The integrity of this barrier is meticulously maintained by cellular adnexin molecules that reside at the endothelial cell junctions, including VE-cadherin and claudin/occludin (133). Research has substantiated that the incursion of exogenous pathogen-associated molecular patterns (PAMPs) or the release of endogenous damage-associated molecular patterns (DAMPs) trigger the endocytosis and subsequent degradation of VE-cadherin. This process results in the disintegration of the pulmonary microvascular endothelial barrier’s integrity and a rise in vascular permeability, which is a defining characteristic of acute lung injury (ALI) and acute respiratory distress syndrome (ARDS) during sepsis (134–136). Beyond their role as a barrier, endothelial cells (ECs) possess metabolic and secretory functions that are integral to the physiological processes of inflammation, thrombosis, vascular permeability, and fluid balance. During septic ALI/ARDS, ECs demonstrate a remarkable capacity to transition swiftly from a quiescent state to a highly inflammatory and activated one. This is evidenced by an augmented secretion of cytokines, chemokines, and procoagulants, as well as an upregulation of adhesion molecules including intercellular adhesion molecule-1 (ICAM-1), vascular cell adhesion molecule-1 (VCAM-1), and E-selectin. These dynamic processes in ECs are instrumental in the recruitment of leukocytes, the neutralization of bacteria, and the containment of pathogen dissemination (137, 138). However, an overly exuberant cellular response can precipitate excessive activation of the complement system, aberrant immune responses, inflammatory storms, and coagulation disorders—pathological changes that further exacerbate the damage to pulmonary microvascular endothelial cells and precipitate diffuse pulmonary microvascular thrombosis. Under physiological conditions, the primary mode of energy supply for endothelial cells (ECs) mirrors that of tumor cells, with glycolysis taking precedence over oxidative phosphorylation (15). Upon inflammatory stimulation, such as that induced by lipopolysaccharide (LPS), the glycolytic activity in endothelial cells (ECs) is further intensified. This escalation in glycolysis exacerbates the body’s inflammatory response and results in the accumulation of lactic acid, the end product of glycolysis. The lactic acid-induced phosphorylation of extracellular signal-regulated kinase 2 (ERK2) can facilitate the dissociation of ERK2 from VE-cadherin. Consequently, the vascular endothelial cadherin complex on the endothelial cell surface is compromised, leading to heightened vascular permeability (139). Besides, lactate reduced the expression of heat shock proteins, which are required for VE-cadherin-mediated tight junctions in ECs (140). This initiates a vicious cycle. Recent research has demonstrated that PFKFB3, a pivotal activator of glycolysis, is markedly upregulated in both LPS-treated human pulmonary artery endothelial cells (HPAECs) and LPS-stimulated mouse lung tissue ECs. The knockdown of PFKFB3 significantly mitigated LPS-induced glycolysis in HPAECs. Mice with endothelium-specific PFKFB3 deficiency or inhibition exhibited reduced endothelial permeability and pulmonary edema, as well as an enhanced survival rate following LPS challenge compared to wild-type mice. This improvement was concurrent with a downregulation of ICAM-1 and VCAM-1 expression, and a diminished infiltration of neutrophils and macrophages in the lung tissue (131, 141). It has been observed that Shenfu injection effectively mitigates LPS-induced inflammatory activation and the associated increase in endothelial cell permeability by targeting the PI3K/AKT-mediated glycolytic pathway (142). Pyruvate dehydrogenase (PDC) facilitates the conversion of pyruvate to acetyl-CoA, thereby promoting the tricarboxylic acid (TCA) cycle. According to Mao et al. (143), the activation of the PDC complex counteracts the LPS-induced enhancement of glycolysis in vascular endothelial cells. This results in decreased lactate production, reduced vascular hyperpermeability, improved endothelial function, and a hastened rate of aerobic oxidation. Interestingly, the activated PDC complex is metabolized to lactate by lactate dehydrogenase (LDH), a biomarker used to assess the severity of sepsis, in the final step of glycolysis. In essence, glycolysis is upregulated in endothelial cells (ECs) with sepsis-induced lung injury, and the accumulation of lactate can precipitate hyperpermeability and endothelial dysfunction (144). Curbing the aberrant glycolytic flux in endothelial cells can preserve their integrity and diminish their polarization towards an inflammatory phenotype, thereby potentially improving the prognosis of sepsis-induced lung injury.

#### 3.2.2 Epithelial cells

Beyond endothelial cells, the metabolic regulation of epithelial cells also assumes a significant role in sepsis-induced lung injury. Research in this area primarily concentrates on several key aspects. In the context of sepsis-associated ALI, alveolar type II epithelial cells sustain damage, resulting in a reduction of alveolar surfactant secretion, alveolar collapse, and an exacerbation of pulmonary edema. Throughout the lung injury repair process, type II epithelial cells are capable of differentiating into type I epithelial cells, thus preserving a healthy cell population and forestalling the advancement of lung injury to ARDS (145). Furthermore, the interplay between oxidative stress and metabolic regulation has captured the attention of numerous researchers. The overproduction of reactive oxygen species (ROS) or a deficiency in antioxidant enzymes can disrupt lipid, protein, and nucleic acid metabolism, leading to an imbalance between pro-oxidant and antioxidant systems and causing harm to the body. Excessive ROS can trigger ALI, inflict cellular damage, activate pro-apoptotic signaling pathways, and ultimately lead to the demise of alveolar epithelial and endothelial cells. Nuclear factor-κB (NF-κB) is a pivotal target in lung tissue damage induced by oxygen free radicals. Under stress, NF-κB can initiate the transcription of key cytokines such as tumor necrosis factor-α (TNF-α), interleukin-6 (IL-6), and interleukin-1β (IL-1β), and can also stimulate the activation of inflammatory cells (146, 147). Building on previous discussions, the role of neutrophil extracellular traps (NETs) in sepsis-induced acute lung injury (SI-ALI) has been further explored. Studies have revealed that NETs contribute to SI-ALI by exacerbating ferroptosis in alveolar epithelial cells. The overabundance of NETs correlates with an upregulation of ferroptosis, a process that hinges on the METTL3-catalyzed N6-methyladenosine (m6A) modification of hypoxia-inducible factor-1α (HIF-1α), followed by a reprogramming of mitochondrial metabolism (148–151). Moreover, the heme oxygenase-1/carbon monoxide (HO-1/CO) system is emerging as a potential novel endogenous protective mechanism that modulates cellular endoplasmic reticulum stress (ERS). The activation of the HO-1/CO system has been shown to suppress ERS and apoptosis in lung tissue cells, thereby ameliorating ALI in septic mice. This system mediates its protective effects by curbing ERS and diminishing lipopolysaccharide (LPS)-induced oxidative stress injury in alveolar macrophages (152–154). Ultimately, it is crucial to focus on the shifts in cellular metabolism. The influence of cellular metabolism, as modulated by the pulmonary microenvironment, has been a subject of scrutiny during the course of acute lung injury (155, 156). For instance, autophagy is increasingly recognized as an alternative form of programmed cell death. Deficiencies or reductions in autophagy levels, along with the inhibition of autophagosome degradation, are implicated in the pathogenesis of ALI in the context of sepsis. Autophagy thus emerges as a promising therapeutic target for mitigating inflammatory lung tissue injury associated with sepsis.

## 4 Regulatory mechanisms related to glycolytic reprogramming in sepsis-induced lung injury

### 4.1 Traditional signaling pathways

**Figure 3:**
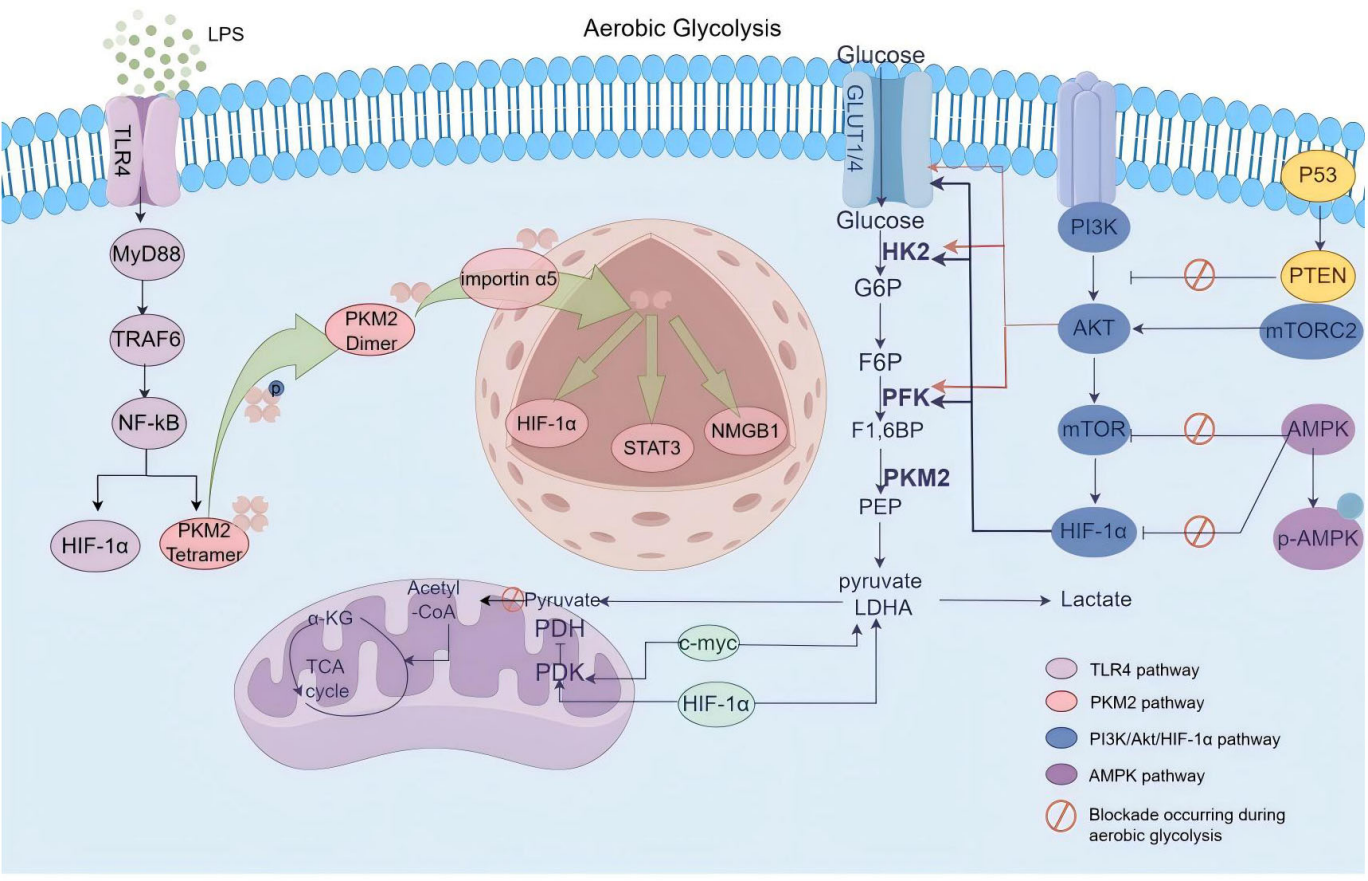
Schematic overview of signaling pathways governing glycolytic metabolic reprogramming. In the context of ALI/ARDS, the metabolic reprogramming of pertinent immune cells is characterized by the intricate interplay and regulation of various signaling cascades. Pathways including TLRs, PI3K-Akt-mTOR, and PKM2-HIF-1α are triggered by inflammatory stimuli, which in turn enhance the expression of pivotal glycolytic enzymes and facilitate metabolic reprogramming. Under physiological conditions, the AMPK pathway typically acts to restrain glycolytic metabolism reprogramming; however, in sepsis-induced lung injury, this suppressive effect is compromised, thereby impacting the prognosis of ALI/ARDS.

In the context of ALI/ARDS, the metabolic reprogramming of key immune cells is marked by a complex interplay and regulation of diverse signaling pathways. Pathways such as Toll-Like Receptors (TLRs), Phosphoinositol-3 Kinase-Protein Kinase B-mammalian Target of Rapamycin (PI3K-Akt-mTOR), and Pyruvate Kinase M2-Hypoxia-inducible factor 1-alpha (PKM2-HIF-1α) are activated by inflammatory stimuli, which subsequently amplify the expression of crucial glycolytic enzymes and promote metabolic reprogramming (**Figure 3**).

#### 4.1.1 Toll-like receptor pathways

In recent years, a growing body of evidence has highlighted the pivotal role of Pattern Recognition Receptors (PRRs) in the activation of innate immunity during sepsis-induced lung injury. Upon infection, PRRs initiate the activation of immune cells by detecting Pathogen-Associated Molecular Patterns (PAMPs) or Damage-Associated Molecular Patterns (DAMPs), thereby orchestrating an inflammatory response (157–159). Toll-like Receptors (TLRs) represent a significant subset of PRRs, comprising type I transmembrane proteins. A total of thirteen TLRs have been identified in mammals, with humans possessing ten (TLR1-10) and mice twelve (TLR1-9, TLR11-13). Among these, TLR4 has been the subject of the most extensive research (160). TLR4 expression is detectable across a spectrum of immune cells, endothelial cells, and alveolar epithelial cells, where it orchestrates a series of reactions that govern cellular metabolic alterations and exerts a pivotal influence on the progression of sepsis-induced lung injury (161). TLR4 forms a heterodimer with Myeloid Differentiation Factor 2 (MD2). Lipopolysaccharide (LPS) is facilitated by Lipopolysaccharide Binding Protein (LBP) to bind with CD14, and subsequently, it is recognized by the TLR4/MD2 complex. This interaction triggers the activation of downstream signaling pathways, encompassing both Myeloid Differentiation Factor 88 (MyD88)-dependent and MyD88-independent cascades, which in turn regulate the release of a spectrum of inflammatory mediators (162). Overabundance of inflammatory factors compromises the integrity of tight junctions between alveoli and capillaries, augmenting vascular permeability and prompting the seepage of protein-rich exudate into the alveolar spaces. This results in impaired gas exchange and the onset of pulmonary edema. Concurrently, TLR4 activation instigates swift metabolic shifts in immune cells, such as macrophages and dendritic cells, which are notably characterized by escalated aerobic glycolysis and diminished oxidative phosphorylation. This leads to the accumulation of tricarboxylic acid (TCA) cycle metabolites, including citrate and succinate. The TLR4-driven glycolytic reprogramming is pivotal for the survival and functionality of immune cells, as it fulfills the heightened energy and nutrient requirements associated with rapid activation and the synthesis of effector molecules (163, 164).

Researchers have employed metabolic tracing and mass spectrometry to demonstrate that under LPS stimulation, TLR signaling induces cellular metabolic reprogramming. This process increases the availability of acetyl-CoA derived from glucose and subsequently enhances histone acetylation. The adaptor proteins MyD88 and TRIF activate ATP-citrate lyase, which in turn, facilitates the expression of a variety of gene sets induced by LPS (165). Research indicates that TLR4 expression is markedly upregulated in septic rats, coincident with heightened expression of glycolysis-associated proteins such as HIF-1α and PKM2. The Xijiao Dihuang decoction ameliorates sepsis prognosis by curbing glycolysis, achieved through the inhibition of TLR4 and its downstream signaling pathways (166). Li (167) found that Norbergenin inhibits glycolysis by suppressing TLR-mediated downstream NF-κB, MAPK, and STAT3 signaling pathways, blocking metabolic reprogramming, promoting mitochondrial oxidative phosphorylation, and restoring abnormal metabolites in the TCA cycle to prevent LPS-induced macrophage inflammatory responses. Intriguingly, TLR agonists have been demonstrated to initiate immune stress via metabolic reprogramming and epigenetic alterations, prompting premature glycolytic reprogramming and activation in dendritic cells, which in turn substantially bolsters their antibacterial capabilities (163, 168). Consequently, strategically promoting or inhibiting TLR activation at the right moments, along with modulating the shift of immune cell metabolism from oxidative phosphorylation to aerobic glycolysis, could present novel therapeutic avenues for the treatment of sepsis.

#### 4.1.2 AMPK pathway

AMP-activated protein kinase (AMPK) is a highly conserved heterotrimeric protein kinase complex, consisting of a catalytic α subunit and two regulatory β and γ subunits (169). AMP-activated protein kinase (AMPK), serving as the principal sensor of cellular energy balance in mammals, plays an essential role in the regulation of glucose and lipid metabolism. When the body’s metabolic activity is heightened and energy expenditure surges, AMPK becomes phosphorylated and activated. It fosters oxidative phosphorylation and fatty acid β-oxidation by modulating the expression of rate-limiting metabolic enzymes and the activity of epigenetic regulators. However, during sepsis, the AMPK pathway is suppressed, resulting in an increased glycolytic capacity in immune cells, such as macrophages. This shift directly influences the secretion of inflammatory and anti-inflammatory cytokines, including IL-1β and IL-6 (170). Mechanistically, the lack of AMPK increases the PKM2-dependent aerobic glycolysis, leading to increased release of the late mediator of lethal systemic inflammation, high-mobility group box 1 (HMGB1), from macrophages and monocytes (171). Moreover, research has established that macrophage Sprouty4 (Spry4) and SAMSN1 modulate sepsis-induced lung injury via the AMPK pathway. The overexpression of macrophage Spry4 intensifies inflammation, oxidative stress, and lung injury in septic mice, whereas Spry4 deficiency mitigates sepsis-induced lung injury by activating the CaMKK2/AMPK pathway. In contrast, the overexpression of macrophage SAMSN1 ameliorates LPS-induced sepsis lung injury in mice. This improvement is primarily attributed to SAMSN1’s binding to growth factor receptor-bound protein 2-associated protein 1 (GAB1), which prevents its degradation, subsequently enhancing PKA/AMPKα2 activation in a PTPN11 (also known as SHP2)-dependent manner (172, 173). Thus, the AMPK pathway plays an important regulatory role in macrophages’ response to sepsis-induced lung injury.

Recognizing the pivotal role of glycolytic metabolism in sepsis-induced organ injury, the therapeutic administration of 2-deoxy-D-glucose (2-DG) to inhibit glycolysis has been shown to upregulate SIRT3 and p-AMPK expression, thereby significantly ameliorating sepsis-induced renal injury. In contrast, the glycolytic byproduct lactate downregulates SIRT3 and p-AMPK expression, aggravating the injury. The lactate/SIRT3/AMPK pathway, by inhibiting aerobic glycolysis, alleviates sepsis-induced kidney injury, indicating a potential reclassification of lactate from a “metabolic waste” to a therapeutic target for organ damage in sepsis. Furthermore, AMPK activators such as metformin and limonin have been demonstrated to inhibit HIF-1α expression in mice, thereby reducing aerobic glycolysis and moderating sepsis-related inflammatory responses (174, 175). Growth differentiation factor 15 (GDF15), recognized as a stress response cytokine, mitigates sepsis-induced lung injury through the promotion of AMPK phosphorylation, inhibition of glycolysis in alveolar macrophages, and attenuation of NF-κB/MAPKs signaling (176). Tangeretin, on the other hand, diminishes sepsis-induced lung injury by modulating the PLK1/AMPK/DRP1 signaling axis, thereby inhibiting ROS-mediated NLRP3 inflammasome activation (177). Quercetin mitigates sepsis-induced lung injury by dampening oxidative stress-mediated endoplasmic reticulum (ER) stress through the activation of SIRT1/AMPK pathways (178). Consequently, AMPK emerges as a negative regulator of bioenergetic reprogramming in immune cells and stands out as a promising target for therapeutic intervention in sepsis.

#### 4.1.3 PI3K/Akt/mTOR pathway

The PI3K/Akt/mTOR signaling pathway is a canonical axis that fosters cell proliferation, metabolism, and angiogenesis. It is also a pivotal regulatory pathway implicated in the Warburg effect observed in sepsis and exerts a significant influence on sepsis-induced lung injury and pulmonary fibrosis (179, 180). The signaling enzyme PI3K is comprised of a regulatory subunit p85 and a catalytic subunit p110, harboring both phosphatidylinositol kinase and protein kinase activities. Upon external stimulation, PI3K is activated, converting phosphatidylinositol bisphosphate (PIP2) into phosphatidylinositol trisphosphate (PIP3), which subsequently activates downstream signaling molecules such as the protein kinase Akt. Activated Akt boosts GLUT1 expression, thereby promoting glucose uptake in T lymphocytes. Additionally, Akt, once activated, upregulates HIF-1α expression through mTOR, prompting immune cells to redirect glucose metabolism towards glycolysis even under aerobic conditions (181). As a cellular energy regulator, mTOR plays a critical role in maintaining cellular energy homeostasis (182). mTOR exists within two distinct multiprotein complexes, mTORC1 and mTORC2. The mTORC1/HIF-1α signaling pathway is capable of promoting aerobic glycolysis in macrophages, guiding them towards an inflammatory M1 phenotype. In contrast, mTORC2 facilitates the polarization of macrophages towards an anti-inflammatory M2 phenotype (183–185). Investigations by Pan and colleagues have revealed that the PI3K/Akt-HIF-1α pathway governs the expression of LDHA and influences the immune functionality of neutrophils (186). The pathway inhibitor LY294002 substantially decreases the phosphorylation levels of PI3K and Akt, as well as the protein expression levels of HIF-1α and LDHA. Moreover, LY294002 significantly suppresses lactate production, neutrophil chemotaxis, and phagocytosis. This suggests that inhibiting the PI3K/Akt axis can markedly reduce glycolytic activity in neutrophils during sepsis. A growing body of evidence underscores the pivotal role of the PI3K/Akt/mTOR signaling pathway in the etiology and progression of septic lung injury, mediated through immune cell metabolic reprogramming. LPS stimulates aerobic glycolysis and curbs autophagy by activating the PI3K/Akt/mTOR-PFKFB3 pathway, which in turn, fosters collagen synthesis in lung fibroblasts (180, 187, 188). Furthermore, the PI3K/Akt pathway exerts control over mitochondrial dynamics and provides relief from septic lung injury through the activation of heme oxygenase-1 (HO-1). Additionally, SRPK1 and Songorine are capable of reducing oxidative stress-related inflammation via the same pathway, thereby mitigating LPS-induced purulent acute lung injury (189–191). The PI3K/Akt signaling pathway also plays a role in enhancing erythropoietin (EPO)-mediated endotoxin-tolerant macrophage function and metabolic reprogramming by upregulating the expression of Irak3 and Wdr5. This upregulation contributes to reduced lung injury, improved bacterial clearance, and diminished mortality in LPS-tolerant mice following secondary infection (192). Recently, traditional Chinese medicines (such as Shenfu injection, Rhodiola rosea, and ginsenoside Rg1) have also been shown to exert protective effects against septic lung injury through the PI3K/Akt pathway (193–195). These studies underscore that the PI3K/Akt/mTOR signaling pathway possesses substantial clinical translational potential, serving as a therapeutic target for enhancing immune function in sepsis.

#### 4.1.4 PKM2-HIF-1α pathway

Pyruvate kinase (PK) is a pivotal rate-limiting enzyme in the glycolytic pathway, responsible for transferring a phosphate group from phosphoenolpyruvate (PEP) to ADP, resulting in the production of pyruvate and ATP (196). Pyruvate kinase is expressed in four isoforms: PKM1, PKM2, PKL, and PKR. Prominent among them, PKM2 is a multifunctional enzyme that engages in both metabolic and non-metabolic activities, exerting a significant influence on the metabolism of inflammatory and immune cells as well as the body’s inflammatory response. PKM2 is capable of sensing intracellular and extracellular regulatory signals through its interaction with various effectors. It dynamically shifts between a high-activity tetramer and a low-activity dimer state through allosteric regulation, thereby modulating its metabolic activity (197, 198). Under resting conditions, PKM2 primarily exists as a tetramer in the cytoplasm, where it performs its role as a metabolic kinase, catalyzing the conversion of phosphoenolpyruvate to pyruvate (199). Upon LPS stimulation, ROS-mediated oxidative modification targets the cysteine residue C358 in immune cell PKM2. In addition to this, modifications such as succinylation, acetylation, and phosphorylation induce a conformational shift in PKM2 from a high-activity tetramer to a low-activity dimer, facilitating its translocation to the nucleus (200–202). Within the nucleus, the dimeric form of PKM2 interacts with factors such as HIF-1α, HMGB1, and STAT3, thereby promoting the transcription of glycolytic enzymes including GLUT1, LDHA, and HK. This interaction redirects the glucose metabolism of immune cells from oxidative phosphorylation to aerobic glycolysis and enhances the release of inflammatory mediators from immune cells (203, 204). Beyond modulating immune cell metabolism to influence the production of inflammatory factors, PKM2 can also directly bind to HIF-1α in the nucleus, thereby promoting the transcription of genes encoding inflammatory factors such as IL-1β. This action initiates an inflammatory response within the body (202). Using PKM2 activators like DASA-58 or TEPP-46 to inhibit PKM2 nuclear translocation can downregulate LPS-induced IL-1βexpression (203). Moreover, the junction protein 4.1R on PKM2 can directly engage with TLR4, thereby inhibiting the activation of the AKT/HIF-1α signaling pathway. This interaction targets HIF-1α-mediated glycolysis and modulates the polarization of M1 macrophages (205). Furthermore, research by Pei and colleagues has demonstrated that PKM2 tetramers can prevent the conversion of PKM2 to its dimeric form, thereby diminishing HIF-1α activation and curbing aerobic glycolysis (206). Recent studies indicate that serum PKM2 levels are significantly elevated in sepsis patients, leading to a higher incidence of organ damage and septic shock (207). Additionally, SENP3 can enhance M1 macrophage polarization and the production of pro-inflammatory cytokines through the HIF-1α/PKM2 axis, contributing to sepsis-induced lung injury. Conversely, Norisoboldine and the flavonoid compound Cynaroside can mitigate sepsis-induced acute lung injury by promoting the polarization of macrophages from M1 to M2 via the PKM2/HIF-1α pathway (206, 208, 209). Hence, the PKM2/HIF-1α axis serves as a nexus between the metabolic reprogramming of immune cells and the inflammatory response. Modulating HIF-1α-driven immune metabolism via PKM2 offers new potential targets for curbing uncontrolled inflammatory responses in the context of sepsis-induced lung injury.

### 4.2 Epigenetic regulatory mechanisms

**Figure 4:**
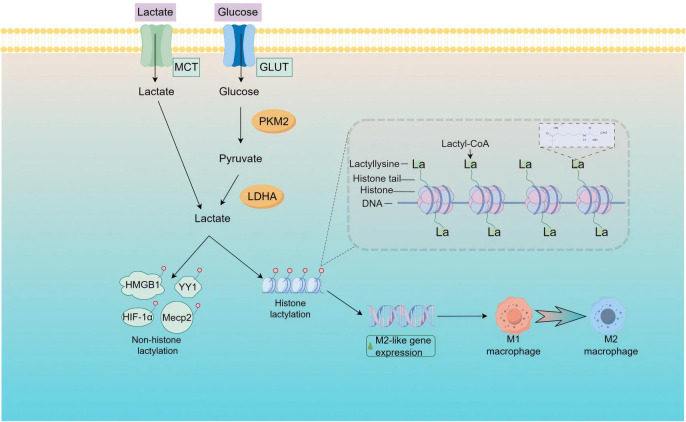
Schematic representation of lactylation modifications in inflammatory contexts. Lactate, predominantly generated intracellularly through glycolysis, can also be imported from the extracellular environment into the cell via the MCT1 transporter. Upon accumulation within the cell, lactate is transformed into lactyl-CoA, which then catalyzes epigenetic regulation through the lactylation of specific histones and non-histone proteins.

Inflammation and epigenetics have emerged as prominent areas of focus within the realm of biomedical research. Epigenetics encompasses the study of genetic changes that occur without alterations to the underlying DNA sequence, encompassing DNA methylation, histone modifications, and non-coding RNAs. An increasing body of research has highlighted the intricate interplay between inflammation and epigenetics. Epigenetic modifications are subject to extensive regulation during the progression of sepsis and are pivotal in the immunosuppression observed in the later stages of the condition (210). Oxygen sensing and energy metabolism are widely involved in various physiological and pathological processes. For instance, the Warburg effect, a special energy metabolism characteristic in tumors, involves a preference for glycolysis to obtain energy even in the presence of oxygen, resulting in a significant accumulation of lactate (211, 212). Recent research has transformed the perception of accumulated lactate from being simply a cellular energy source and metabolic byproduct to a molecule with substantial regulatory functions in biological processes. As shown in **Figure 4**, lactate, which accumulates during metabolism, can serve as a precursor for lactylation modifications and plays a role in the homeostatic regulation of M1 macrophages during bacterial infection. These findings not only pave new paths for exploring post-translational protein modifications but also offer novel perspectives on understanding the regulatory mechanisms of metabolic byproducts, such as lactate, in immune responses.

In the context of septic lung injury, metabolic reprogramming results in the substantial production of lactate. The accumulation of lactate within cells leads to histone lactylation, a modification that, through its sustained regulation of immune cells, influences immune remodeling during sepsis. This process plays a pivotal role in the progression and prognosis of sepsis (213). Lactylation is a form of post-translational protein modification that involves the attachment of lactate residues to lysine residues on proteins. This covalent alteration can regulate the affinity of histone-DNA binding, modulate the accessibility of gene loci, and thereby influence gene expression. Studies have indicated that metabolic reprogramming, stemming from imbalances in glycolysis and the TCA cycle, results in heightened histone lactylation. Specifically, lactylation of histone H3 lysine 18 (H3K18la) upregulates the expression of genes involved in damage repair and homeostasis, such as arginase 1 (Arg1). This shift aids in the transition of macrophages to an M2 repair phenotype, reduces the levels of pro-inflammatory cytokines, and fosters the secretion of anti-inflammatory cytokines (214, 215). This implies that H3K18la has a role in inflammation-related diseases. In a clinical study focusing on sepsis, the expression of H3K18la was notably higher in peripheral blood monocytes from critically ill patients and showed a positive correlation with the levels of IL-6 and IL-10, as well as with Arg-1 mRNA expression (216). Additional research has shown that lactate modulates the levels of N6-methyladenosine (m6A) modification by binding H3K18la to the METTL3 promoter site. The m6A modification is particularly enriched in ACSL4, which increases its expression and subsequently promotes mitochondrial-related ferroptosis. The knockdown or targeted inhibition of METTL3 effectively mitigates lactate-induced ferroptosis in alveolar epithelial cells and lessens lung injury in septic mice (150). These findings indicate the potential role of H3K18la in the recognition of sepsis and its potential utility as a diagnostic marker or prognostic indicator for septic shock. This could assist in the clinical assessment of patients’ immune status and inform treatment strategies. Intriguingly, metabolic reprogramming in pulmonary myofibroblasts results in increased glycolysis and a more pronounced fibrotic phenotype. Lactate can induce the expression of profibrotic genes (ARG1, PDGFA, THBS1, and VEGFA) in macrophages through lactylation; when the lactylation regulator p300 is downregulated, the fibrotic phenotype is mitigated (217). Lactylation is a modification that occurs ubiquitously in various cellular compartments, including the nucleus, cytoplasm, mitochondria, endoplasmic reticulum, and cytoskeleton. Notably, lactylation at the K147 site of Aldolase A (ALDOA) is a common occurrence; this modification diminishes ALDOA’s activity, thereby inhibiting glycolysis, reducing lactate production, and establishing a negative feedback mechanism to regulate glycolysis (218). PKM2, a crucial regulator of glycolytic reprogramming during sepsis, experiences lactylation at the K62 site. This modification inhibits glycolysis and prompts macrophages to shift towards a reparative phenotype, resulting in the upregulation of Arg-1 expression and, ultimately, facilitating wound healing in mouse wound models (219). HMGB1 is a nuclear protein that plays a crucial role in late immune responses during sepsis (220). Reports suggest that under sepsis conditions, there is an increase in lactylated HMGB1, which is secreted by macrophages via exosomes, thereby enhancing endothelial cell permeability. This indicates that lactylation can impact sepsis not only through histone proteins but also in a non-histone capacity (221). Intriguingly, it has been established that human primary alveolar macrophages (AMs) co-cultured with alveolar epithelial cells exhibit a diminished inflammatory response upon exposure to LPS. The underlying mechanism is likely that the transcription factor hypoxia-inducible factor (HIF)-1α facilitates an increase in glycolysis within alveolar type II epithelial cells (AT II). The lactate, a byproduct of this glycolysis in AT II cells, induces a shift in AMs towards an anti-inflammatory phenotype. This shift results in an upregulation of IL-10 and a downregulation of IL-1 and IL-6 expression in AMs, thereby mitigating acute lung injury (ALI). Mice with a specific deletion of HIF-1α in AT II cells and those treated with lactate dehydrogenase inhibitors displayed exacerbated inflammation and worsened ALI (222). Consequently, glycolysis-related enzymes modulate lactylation modifications by regulating lactate levels, and these enzymes can themselves undergo lactylation, providing a negative feedback on glycolysis. This feedback regulatory loop warrants further exploration and elucidation. Current research suggests that the interplay between metabolic reprogramming and epigenetic changes is intricate and robust. The aforementioned findings underscore the inextricable link between histone and non-histone lactylation modifications and sepsis, and they underscore their potential as pivotal indicators for diagnosing sepsis, gauging disease severity, predicting outcomes, and informing treatment strategies.

## 5 The impact of glucose metabolism reprogramming on the occurrence, development, and prognosis of S-ALI

In the context of septic lung injury, the metabolic reprogramming in immune cells is characterized primarily by a transition from oxidative phosphorylation to aerobic glycolysis. This metabolic shift is pivotal for immune cell activation, proliferation, and differentiation. Rapid metabolic reprogramming supplies the necessary energy and metabolites for immune cell activation, bolsters host tolerance, and aids in preventing the escalation of sepsis to multiple organ dysfunction syndrome (MODS). However, when cells persist in a state of aerobic glycolysis over extended periods, there is a substantial release of inflammatory cytokines, which can lead to a cytokine storm and extensive cellular damage. Moreover, glycolytic byproducts, such as lactate, can induce immune tolerance and influence the prognosis of sepsis (223). Thus, pinpointing and precisely modulating the optimal timing for immune cell metabolic reprogramming is essential for the prevention and treatment of septic lung injury (224).

### 5.1 Metabolic reprogramming promotes activation of immune cells

During the initial phases of sepsis, aerobic glycolysis is advantageous for stimulating the immune system. On one hand, LPS interacts with Toll-like receptors on innate immune cells, thereby activating downstream effector molecules and triggering the upregulation of HIF-1α and GLUT1 expression. The elevated expression of GLUT1 enhances glucose uptake by immune cells (225–227), supplying the requisite energy sources and biosynthetic materials needed for their activation (228). When cells exhibit the Warburg effect, the tricarboxylic acid (TCA) cycle is impeded at the first and third dehydrogenation stages, resulting in the accumulation of citrate and succinate. Citrate plays a role in the synthesis of prostaglandins and nitric oxide, whereas succinate is associated with IL-1β release and the induction of inflammation (58). Metabolic byproducts of aerobic glycolysis, including dihydroxyacetone phosphate (DHAP), glyceraldehyde 3-phosphate (G-3-P), and glucose 6-phosphate (G-6-P), are instrumental in lipid synthesis, amino acid synthesis, and nucleotide synthesis, respectively (33, 34), providing the biosynthetic materials required for immune cell proliferation and differentiation. Conversely, despite metabolic reprogramming and the suppression of OXPHOS, the uptake of glucose and the rate of glycolysis in immune cells persist in increasing, rapidly generating ATP to fulfill the energy demands associated with immune activation. Aerobic glycolysis drives macrophage polarization towards the pro-inflammatory M1 phenotype, with PKM2 facilitating the phosphorylation of EIF2AK2 in macrophages, thereby promoting the activation of NLRP3 and AIM2 inflammasomes. This leads to the release of IL-1β and HMGB1, key mediators in the inflammatory response (229, 230). Additional research indicates that neutrophils, possessing lower mitochondrial content, rely heavily on aerobic glycolysis for their immune function. Neutrophils depend on glycolysis to swiftly produce ATP necessary for the formation of neutrophil extracellular traps (NETs) induced by PMA (231). The inhibition of aerobic glycolysis using 2-deoxyglucose (2-DG) significantly hampers glucose uptake and glycolysis, consequently affecting NET formation (232). Consequently, metabolic reprogramming equips the immune system with ample energy and biosynthetic materials, enabling a more potent and swift immune response.

Simultaneously, as the frontline of pulmonary immunity, alveolar macrophages can initiate an immune memory response known as “trained immunity” following pathogen stimulation. This is characterized by an enhanced release of proinflammatory cytokines from macrophages, epigenetic changes that incline the cells towards an inflammatory phenotype, and a metabolomic shift that favors glycolysis and fatty acid oxidation (233, 234). Recent studies have demonstrated that the inhalation of LPS induces trained immunity in alveolar macrophages (AMs), resulting in increased endocytic efficiency, upregulated transcription of lipid and arginine metabolism, and ultimately enhanced inflammatory responses (235). Another study found that BCG can alter the metabolism and epigenetic regulation of AMs by activating the mTORC2/HK1 signaling pathway, promoting the development of trained immunity and providing protection against severe acute respiratory syndrome coronavirus 2 (SARS-CoV-2) infection. Additionally, the adoptive transfer of trained AMs has shown significant efficacy in reducing sepsis-induced lung injury, underscoring the potential for developing AM-based cell therapies (236, 237). These discoveries could pave the way for a life-saving strategy during the early stages of sepsis-induced lung injury, bridging the gap until appropriate therapeutic interventions can be identified and implemented.

### 5.2 Prolonged glycolysis leads to immune suppression

In the advanced stages of sepsis, immune cells, including macrophages and dendritic cells, undergo excessive activation due to metabolic reprogramming, leading to a torrential release of inflammatory factors. This unleashes an inflammatory mediator storm that severely impairs the structure and function of normal host cells. While many sepsis patients may weather the initial inflammatory storm, the subsequent immune suppression further exacerbates their critical condition (238–240). Immune suppression is primarily characterized by a decrease in the gene expression of pro-inflammatory factors like IL-6 and TNF-4, and monocyte chemotactic factors such as CCL2, while there is an upregulation in the expression of anti-inflammatory factors such as IL-4 and IL-10 (241, 242). The epidermal growth factor receptor (EGFR) facilitates the translocation of GLUT1 to the cell surface via its downstream TBK1/Exo84/RalA protein complex, thereby inducing metabolic reprogramming and apoptosis in CD4+ T lymphocytes. This process impacts immune function, contributes to immune cell exhaustion, and exacerbates immune suppression in sepsis (243). Lactate, a key glycolytic metabolite, plays a pivotal role in the transition from an inflammatory storm to immune suppression. In the context of sepsis, glucose metabolism shifts from oxidative phosphorylation to aerobic glycolysis, leading to the substantial production of lactate by immune cells. Lactate enters the cell nucleus and undergoes histone lactylation modification, thereby regulating the expression of inflammation-related genes. Research has indicated that elevated lactate levels significantly suppress the expression of pro-inflammatory genes, such as IL-1β, IL-6, NOS2, and PTGS2, as well as M1-associated genes like CD38, CD80, and CD86, while promoting the transcription of M2-associated genes, including Arg1, Vegfa, and Mgl1. This process inhibits M1 macrophages and facilitates the transition from the M1 to M2 macrophage phenotype (244, 245).

The lactate receptor GPR81, a member of the G protein-coupled receptor family, also acts as a target mediating the lactate-induced transition of macrophages from the M1 to the M2 phenotype (246–248). Overactivation of M2 macrophages induces an immunosuppressive state, thereby further aggravating septic lung injury. Beyond influencing macrophage polarization, lactate can also suppress the expression of FIP200 and light chain 3-II in T cells, thereby promoting T cell apoptosis (249). Lactate also drives T cell death by obstructing the phosphorylation of p38 signaling proteins, thereby suppressing the production of IFN-γ, TNF-α, and IL-2 (250). Furthermore, lactate reduces the production of IL-17A in Th17 cells and induces the expression of Foxp3, prompting the transcriptional reprogramming of pro-inflammatory Th17 cells towards a regulatory T cell (Treg) phenotype characterized by Foxp3 expression. This shift consequently helps to curb inflammation and autoimmunity driven by these cells (104). Environments rich in lactate can also exert an influence on immune cell behavior by augmenting the regulatory function of myeloid-derived suppressor cells, impeding the differentiation of monocytes into dendritic cells, and diminishing their antigen-presenting capabilities (251, 252). Intracellular lactate uptake leads to the lactylation of HMGB1, which accumulates in proximity to cytoplasmic lysosomes and exerts its effects on endothelial cells through exosomal communication. This interaction perturbs endothelial adhesion and tight junction proteins, escalates endothelial permeability, and results in endothelial barrier dysfunction, thereby facilitating lung inflammatory injury (221).

## 6 Mechanisms of targeting glycolytic metabolism for treating S-ALI

Sepsis-induced lung injury, a prevalent and severe complication of infectious diseases, presents a formidable challenge in clinical emergency care, largely due to its high mortality rate. At present, beyond mechanical ventilation and oxygen therapy, no effective treatments exist to enhance prognosis or decrease patient mortality. Extensive research has highlighted that the modulation of glucose metabolism is pivotal in the onset, progression, and prognosis of sepsis-induced lung injury (253). During sepsis, immune cells exhibit the Warburg effect, rapidly generating ATP to bolster cellular resilience against harsh conditions and mitigate oxidative stress from reactive oxygen species on cells and mitochondria. However, the persistence of glycolysis over extended periods can result in irreversible impairment of cellular and organ functions (254). Consequently, achieving a balance between cellular aerobic glycolysis and oxidative phosphorylation, and pinpointing targets that can swiftly restore OXPHOS function, are crucial for enhancing cell survival rates and improving the prognosis of sepsis-induced lung injury. In the context of sepsis-induced lung injury, key enzymes and regulatory factors within glucose metabolism pathways, including HIF-1α, HK2, PKM2, andPFKFB3, modulate cellular energy metabolism and are implicated in oxidative stress and inflammatory responses, showcasing their considerable promise for sepsis treatment (**Table 2**).

**Table 2:** Mechanisms of targeting glycolytic metabolism for treating S-ALI.

Factors	Mechanisms	Therapeutic implication	References
HIF-1α	Under hypoxic conditions in sepsis, HIF-1α acts as a key upstream molecule in glycolysis, promoting the expression of key glycolytic enzymes such as GLUT1, HK2, and PFKFB3.	HIF-1α can increase glucose metabolism in tracheal epithelial cells and promote apoptosis of alveolar type II (AT-II) cells.	(255, 256)
HK	HK is the key rate- limiting enzyme in the first step of the cellular glycolytic pathway, catalyzing the conversion of glucose to glucose-6- phosphate.	It catalyzes glucose glycolysis and initiates pro-inflammatory responses in macrophages through the ATF4-HIF-1α-HK2-glycolysis axis.	(257–259)
PKM2	PKM2, as the key rate-limiting enzyme in the final step of glycolytic metabolism, catalyzes the conversion of phosphoenol pyruvate and ADP to pyruvate and ATP.	Other key rate-limiting enzymes in the aerobic glycolysis pathway further enhance the glycolytic metabolic rate; they regulate the phosphorylation of EIF2AK2 in macrophages to promote the activation of inflammasomes such as NLRP3 and AIM2.	(203, 260–262)
PFKFB3	PFKFB3, a bifunctional enzyme that regulates glycolysis, allosterically activates the key rate- limiting enzyme PFK-1 by regulating and maintaining the intracellular concentration of fructose- 2,6-bisphosphate, thereby accelerating glycolysis.	The expression of PFKFB3 is significantly increased in sepsis-related cells such as macrophages, neutrophils, endothelial cells, and pulmonary fibroblasts, promoting the activation of aerobic glycolysis and leading to acute lung injury.	(132, 263, 264)

### 6.1 Hypoxia-inducible factor

HIF, as a key regulator of hypoxic adaptive inflammatory responses, plays a crucial role in the metabolic transformation process during sepsis (255–258). HIF is a heterodimer composed of an oxygen-dependent α subunit and a constitutively expressed β subunit. Currently, three α subunit isoforms (HIF-1α, HIF-2α, and HIF-3α) and three β subunit homologs (Arnt1, Arnt2, and Arnt3) have been identified in mammalian genomes (259). Among them, HIF-1α is the most abundantly expressed isoform and plays a role in acute hypoxic responses. In quiescent cells, the oxygen-dependent degradation domain (ODDD) of HIF-1α is hydroxylated by prolyl hydroxylase (PHD), which facilitates the binding of the E3 ubiquitin ligase (von Hippel-Lindau, pVHL). This interaction results in ubiquitination and subsequent proteasomal degradation, thereby keeping HIF-1α expression levels low (260, 261). Under hypoxic conditions prevalent in sepsis, HIF-1α serves as a pivotal upstream molecule in glycolysis. Upon binding with HIF-1β, it translocates to the nucleus and stimulates the expression of key glycolytic enzymes, including GLUT1, HK2, and PFKFB3 (262). Further research affirms that throughout the progression of sepsis-induced acute lung injury, HIF-1α can intensify glucose metabolism in tracheal epithelial cells, induce apoptosis of type II alveolar (AT-II) cells, and expedite the development of ARDS (263). This effect may be related to different signaling pathways regulated by HIF-1α. Studies have found that hypoxia inhibits alveolar epithelial cell proliferation and enhances AT-II cell apoptosis by activating the HIF-1α/HRE axis, involving Bnip3L (264). At the same time, studies have confirmed that acetate improves the killing effect of alveolar macrophages against pathogens through NLRP3 inflammasome and glycolysis-Hif-1α axis to better combat bacterial invasion (265). Consequently, targeting HIF-1α may emerge as a novel therapeutic strategy to mitigate acute lung injury. Studies indicate that lidocaine can curb glycolysis by dampening HIF-1α activity in macrophages, influencing the transcription of genes such as GLUT1 and HK2. This inhibition of macrophage activation and M1 polarization contributes to its anti-inflammatory effects (266). Additionally, in sepsis-induced acute lung injury mice, overexpression of histone methyltransferase (SETD2) inhibits HIF-1α, thereby reducing M1 macrophage polarization and glycolysis (267). Dexmedetomidine can also improve LPS-induced acute lung injury *in vivo* and *in vitro* by maintaining mitochondrial dynamics through the HIF-1α/HO-1 signaling pathway (268). Interestingly, rabeprazole, an effective inducer of HIF-1α, can promote vascular repair and resolution of sepsis-induced inflammatory lung injury through endothelial HIF-1α/FoxM1 signaling (269). Thus, the judicious modulation of HIF-1α expression could potentially serve as a therapeutic entry point for enhancing the treatment of sepsis-induced lung injury.

### 6.2 Hexokinase

Hexokinase is a pivotal rate-limiting enzyme that initiates cellular glycolysis by catalyzing the conversion of glucose into glucose-6-phosphate. Mammalian tissues express four isoforms of hexokinase: HK1, HK2, HK3, and HK4 (270, 271). HK1 and HK2, capable of binding to mitochondria, play a crucial role in catalyzing glycolysis to maintain the overall rate of glucose metabolism and satisfy cellular energy demands. As downstream targets of HIF-1α, hexokinases are implicated in the reprogramming of glucose metabolism during sepsis-induced lung injury. Studies indicate that Krüppel-like factor 14 (KLF14) orchestrates the transcriptional regulation of HK2, thereby influencing glycolysis and the immune function of macrophages. KLF14 deficiency is associated with significantly increased mortality in endotoxemia and sepsis models in mice, whereas the pharmacological activation of KLF14 confers protection against sepsis in these murine models (272). Activating transcription factor 4 (ATF4) binds to the promoter region of HK2 and interacts with HIF-1α, thereby initiating pro-inflammatory responses in macrophages through the ATF4-HIF-1α-HK2-glycolysis axis. Specifically, the knockdown of ATF4 diminishes the levels of pro-inflammatory cytokines in LPS-induced mouse splenic macrophages and serum, partially blocking LPS-induced pro-inflammatory cytokines, lactate accumulation, and glycolytic capacity in RAW264.7 cells. Conversely, the overexpression of ATF4 significantly amplifies the reductions in pro-inflammatory cytokines, lactate secretion, and HK2 expression in LPS-induced tolerant macrophages. Thus, ATF4 serves as a pivotal activator of glycolysis, contributing to pro-inflammatory responses and enhancing immune tolerance in macrophages during sepsis (273). In addition to HK2, HK3 has gained increasing recognition for its pivotal role in the development and prognosis of sepsis. *In vitro* studies demonstrate that HK3 levels escalate in LPS-stimulated lung epithelial cells, with a subcellular localization often observed near the nucleus. The suppression of HK3 expression results in diminished proliferation and glycolytic flux in these cells, accompanied by an intensified inflammatory response, heightened oxidative stress, and increased apoptosis (274). In conclusion, hexokinases emerge as promising new targets for immunotherapeutic interventions in sepsis.

### 6.3 Pyruvate kinase M2

Pyruvate kinase M2 (PKM2), one of the four isoforms of pyruvate kinase, stands out as a key rate-limiting enzyme in the culminating step of glycolysis. It facilitates the conversion of phosphoenolpyruvate and ADP into pyruvate and ATP, thereby playing a substantial role in the progression of sepsis (275–277). PKM2 orchestrates the HIF-1α-driven activation of other pivotal rate-limiting enzymes within the aerobic glycolysis pathway, thereby amplifying the rate of glycolysis and resulting in increased lactate and HMGB1 production (26, 203). Furthermore, PKM2-driven glycolysis advances the activation of inflammasomes, including NLRP3 and AIM2, in macrophages through the regulation of EIF2AK2 phosphorylation. This activation subsequently promotes the release of inflammatory cytokines such as IL-1β, IL-18, and HMGB1 from macrophages. Inhibition of the PKM2-EIF2AK2 pathway can safeguard mice from fatal endotoxemia and a variety of microbial sepsis, thereby reducing mortality rates (278). Celastrol (Cel) is a bioactive natural product extracted from the Thunder God vine. It inhibits PKM2 activity by binding to Cys424, thereby suppressing aerobic glycolysis. Additionally, Cel binds to Cys106 in HMGB1, which reduces IL-1β secretion and alleviates the inflammatory response in sepsis (28). Sphingosine kinase 1 (SphK1) interacts with PKM2, facilitating the phosphorylation and nuclear translocation of PKM2. Inhibitors of SphK1 can stabilize PKM2, curb its phosphorylation, and impede its nuclear translocation, consequently leading to a significant reduction in pyroptosis-related markers and mitigating sepsis-induced lung injury (279). The glycolysis inhibitor 2-deoxyglucose (2-DG) alleviates LPS-induced TNF-α and IL-6 release by inhibiting the nuclear PKM2-STAT3 pathway, reducing lung tissue abnormalities, and improving survival rates in LPS-stimulated mice (280). Capsaicin alleviates inflammation and the Warburg effect in sepsis by targeting PKM2-LDHA and COX-2 in a TRPV1-independent manner (281). Furthermore, quercetin diminishes NLRP3 inflammasome activation by impeding PKM2 nuclear accumulation and enhancing SIRT1 expression, thereby protecting against LPS-induced lung injury in mice. Conversely, the SIRT1 inhibitor EX527 escalates NLRP3 activation and PKM2 nuclear accumulation, effectively reversing the anti-inflammatory effects mediated by quercetin (282). Specifically in the context of alveolar macrophages, ephedrine can mitigate LPS-induced glycolysis and M1 polarization by modulating PKM2, thereby alleviating lung injury (283). These findings underscore the pivotal role of PKM2 in immune metabolism and inform the development of targeted treatment strategies for sepsis.

### 6.4 Fructose-2,6-bisphosphatase 3

Fructose-2,6-bisphosphatase 3 (PFKFB3), one of the four isoforms of PFKFB, functions as a bifunctional enzyme in the regulation of glycolysis. It allosterically activates the key rate-limiting enzyme of glycolysis, 6-phosphofructo-1-kinase (PFK-1), by modulating and sustaining intracellular levels of fructose-2,6-bisphosphate. This action, in turn, accelerates the rate of glycolysis (284). Research has revealed that under physiological conditions, PFKFB3 is expressed at low levels across all tissues. However, upon LPS stimulation, PFKFB3 levels markedly increase in cells pertinent to sepsis, including macrophages, neutrophils, endothelial cells, and lung fibroblasts. This elevation promotes the activation of aerobic glycolysis, contributing to the development of acute lung injury (132, 284, 285). Long noncoding RNA GSEC has been shown to bolster neutrophil inflammatory activation by sustaining PFKFB3-involved glycolytic metabolism in the context of sepsis (286). Moreover, PFKFB3 intensifies the formation of neutrophil extracellular traps (NETs), thereby exacerbating sepsis-induced acute lung injury (287).

Transcription factor Zhx2 enhances PFKFB3 transcription by binding to the PFKFB3 promoter, thereby contributing to macrophage metabolic reprogramming and accelerating sepsis progression. The deficiency of Zhx2 in macrophages confers tolerance to LPS-induced glycolysis. In mice with myeloid-specific Zhx2 knockout, there is greater resistance to cecal ligation and puncture as well as LPS-induced sepsis, manifested by prolonged survival, reduced lung injury, and decreased levels of pro-inflammatory cytokines and lactate (288). Additionally, the MAPK family plays a crucial role in the immune response during microbial infections. MAPK phosphatase-1 (MKP-1) functions as a feedback control mechanism for the JNK/p38 MAPK signaling pathway through its dephosphorylation activity. The deficiency of Mkp-1 significantly enhances the expression of PFKFB3, selectively suppresses p38 MAPK activity, but not JNK MAPK, thereby inhibiting PFKFB3 expression and lactate production (289). It is evident that upregulation of PFKFB3 is closely associated with excessive inflammation and high mortality in sepsis (141). Previous studies have reported that LPS drives aerobic glycolysis and collagen synthesis in lung fibroblasts via activation of the PI3K-Akt-mTOR/PFKFB3 signaling pathway (187). Inhibition of PFKFB3 by the inhibitor 3PO or knockdown of the PFKFB3 gene suppresses glycolysis levels and attenuates the differentiation of lung fibroblasts into myofibroblasts (290). Furthermore, 3PO treatment significantly improves survival rates in CLP-induced sepsis mice, partially restores tissue pathology, lung inflammation, lactate increase, and lung apoptosis (291). Additionally, downregulation of PFKFB3 expression can inhibit macrophage pyroptosis and inflammation during sepsis, thus suppressing sepsis development (292). Therefore, targeting PFKFB3-driven glycolysis may be a viable approach for treating septic lung injury.

### 6.5 Others

In addition to the mainstream targets for regulating glucose metabolism in the treatment of sepsis-induced lung injury mentioned earlier, an expanding array of therapeutic targets is being progressively identified and investigated. Studies have reported that exosomes derived from adipose-derived mesenchymal stem cells (ADMSC-Exos) are capable of mitigating acute lung injury induced by sepsis, suppressing inflammation, and stimulating the secretion of TGF-β by macrophages in cecal ligation and puncture (CLP) mice. The silencing of TGF-β has been shown to reverse the protective effects of ADMSC-Exos on sepsis-induced lung injury (293). Additionally, other studies have shown that TGF-β increases the expression and activity of liver-type phosphofructokinase, promotes glycolysis through the mTOR-c-MYC-dependent pathway, and inhibits the activation of transcriptional coactivator SMAD3 and pro-inflammatory transcription factors AP-1, NF-κB, and STAT1, thereby reducing the production of pro-inflammatory cytokines (294, 295). Thus, TGF-β plays a regulatory role in glycolysis within macrophages and independently influences survival in sepsis-induced lung injury models, regardless of the production of inflammatory cytokines. ADMSC-Exos are capable of transferring mitochondrial components from stem cells to alveolar macrophages in a dose-dependent manner. This process enhances macrophage mitochondrial integrity and oxidative phosphorylation levels, facilitates the transition of macrophages to the anti-inflammatory M2 phenotype, restores metabolic and immune homeostasis in airway macrophages, and effectively alleviates lung inflammation and associated pathology (296). The transient receptor potential vanilloid 4 (TRPV4), as a mechanosensitive cation channel, regulates glycolysis in a stiffness-dependent manner by increasing macrophage GLUT1-mediated glucose uptake, thus mitigating sepsis-induced lung injury (297). Additionally, ATP-citrate lyase (ACLY) and angiotensin 17 (Ang-17) have been confirmed to modulate LPS-induced inflammation through regulating the metabolism of sepsis-related cells (298, 299).

Previous research has elucidated that disruptions in glucose metabolism are pivotal in the onset and progression of lung injury induced by sepsis. The intricate nature of glucose metabolism regulation in the body renders current research fragmentary, which spurring investigators to delve into novel therapeutic targets associated with the regulation of glucose metabolism. Future inquiries should augment the exploration of the interplay between glucose metabolism and other metabolic pathways, such as lipid and amino acid metabolism, to achieve a more holistic understanding of the multifaceted mechanisms underlying sepsis. Moreover, clinical trials are essential to substantiate the tangible effects of modulating glucose metabolism on sepsis-induced lung injury, thereby enhancing its clinical applicability. Developing personalized treatment strategies will also emerge as a critical direction for future research, aiming to improve treatment outcomes across diverse patient cohorts. In summary, targeting glucose metabolism furnishes fresh perspectives and therapeutic possibilities for addressing sepsis-induced lung injury. While current studies have underscored its potential clinical utility, further investigation and validation are imperative. Anticipated future research is expected to unravel additional mechanistic intricacies and to refine treatment approaches in this domain, offering more efficacious treatment options for sepsis patients.

## 7 Conclusions and perspectives

The reprogramming of glucose metabolism is instrumental in the progression of septic lung injury and presents novel therapeutic avenues. Glucose metabolism, coupled with its metabolic byproducts and pivotal rate-limiting enzymes, not only engages in inflammatory responses via assorted molecular signaling pathways but also modulates endothelial cell barriers, thereby influencing lung infection processes. Transient glucose metabolism reprogramming can bolster cellular resilience under adverse conditions, mitigate oxidative damage from oxidative phosphorylation, and prolong cell viability. However, sustained excessive glycolysis results in the accumulation of substantial metabolic byproducts, such as lactic acid, inducing immune tolerance and detrimental effects on the prognosis of septic lung injury. Consequently, the timing for manipulating cellular metabolic reprogramming is paramount.

Over the past decade, more than 5,000 studies on septic biomarkers have been published worldwide, identifying 258 distinct biomarkers. Chinese researchers have contributed to this field with several studies of their own; however, systematic validation remains limited, and some controversies persist among these studies, warranting further investigation and synthesis. Future research will concentrate on addressing key challenges, including: (1) unraveling the molecular pathological mechanisms underlying septic lung injury, deciphering the specific regulatory mechanisms of glucose metabolism in septic lung injury, and pinpointing key regulatory factors for adaptive metabolic modulation; (2) developing biomarkers for early diagnosis and prognosis assessment of sepsis, primarily by analyzing metabolic products under pathological and physiological conditions; (3) achieving precise classification and personalized treatment of sepsis. The integration of multidisciplinary approaches, big data analytics, and artificial intelligence will facilitate the early detection and precise treatment of sepsis. In conclusion, the field of immune metabolism and its reprogramming mechanisms opens new avenues for exploring the pathogenesis of sepsis, while targeting glucose metabolism regulation offers fresh hope for the prevention and treatment of septic lung injury. The anticipated future advancements in leveraging immune metabolism to combat septic lung injury are highly anticipated.
